# Children Undergoing Radiotherapy: Swedish Parents’ Experiences and Suggestions for Improvement

**DOI:** 10.1371/journal.pone.0141086

**Published:** 2015-10-28

**Authors:** Charlotte Ångström-Brännström, Gunn Engvall, Tara Mullaney, Kristina Nilsson, Gun Wickart-Johansson, Anna-Maja Svärd, Tufve Nyholm, Jack Lindh, Viveca Lindh

**Affiliations:** 1 Department of Nursing, Umeå University, Umeå, Sweden; 2 Department of Women´s and Children´s Health, Uppsala University, Uppsala, Sweden; 3 Department of Public Health and Caring Sciences, Uppsala University, Uppsala, Sweden; 4 Umeå Institute of Design, Umeå University, Umeå, Sweden; 5 Section of Oncology, Department of Oncology, Radiology and Clinical Immunology, Uppsala University Hospital, Uppsala, Sweden; 6 Department of Oncology, Radiumhemmet, Karolinska University Hospital, Stockholm, Sweden; 7 Department of Radiation Sciences, Umeå University, Umeå, Sweden; National Cancer Institute, UNITED STATES

## Abstract

Approximately 300 children, from 0 to 18 years old, are diagnosed with cancer in Sweden every year. Of these children, 80–90 of them undergo radiotherapy treatment for their cancer. Although radiotherapy is an encounter with advanced technology, few studies have investigated the child’s and the parent’s view of the procedure. As part of an ongoing multicenter study aimed to improve patient preparation and the care environment in pediatric radiotherapy, this article reports the findings from interviews with parents at baseline. The aim of the present study was twofold: to describe parents’ experience when their child undergoes radiotherapy treatment, and to report parents’ suggestions for improvements during radiotherapy for their children. Sixteen mothers and sixteen fathers of children between 2–16 years old with various cancer diagnoses were interviewed. Data were analyzed using content analysis. The findings showed that cancer and treatment turns people’s lives upside down, affecting the entire family. Further, the parents experience the child’s suffering and must cope with intense feelings. Radiotherapy treatment includes preparation by skilled and empathetic staff. The parents gradually find that they can deal with the process; and lastly, parents have suggestions for improvements during the radiotherapy treatment. An overarching theme emerged: that despair gradually turns to a sense of security, with a sustained focus on and close interaction with the child. In conclusion, an extreme burden was experienced around the start of radiotherapy, though parents gradually coped with the process.

## Introduction

Every year, approximately 300 children between 0–18 are diagnosed with cancer in Sweden [1]. Depending on their diagnosis, treatment may include chemotherapy, surgery, radiotherapy, or some combination of these. According to data from the Swedish quality assurance registry for pediatric radiotherapy (RADTOX), 80–90 children were treated with radiotherapy each year during 2008–2013 (unpublished). Children undergoing cancer treatment often experience pain, fear and worry due to the disease, treatment-related pain, and side-effects of drugs [2]. They also experience stressors related to cancer treatment and side-effects, including pain, hair loss and blood tests, and they have to endure invasive and strenuous treatments [3].

Parents of children diagnosed with cancer have described the impact of diagnosis and treatment as devastating and associated with feelings of shock, upset, anger and stress. Furthermore, they express sorrow, anxiety and feelings of uncertainty about their child’s prognosis [4–6]. Parents often want to be close to their child, doing all they can, watching over and acting on behalf of their child, comforting and supporting [7], and their care burden increases [6]. Parents play an important, but emotionally demanding, role during this process. In moments of despair, meaningful activities like caring for the child can lessen the suffering. Parents struggle to view treatment with optimism and hope, while the question of their child’s survival is ever-present. They fear the death of their child [4, 5].

Research has shown that when a child is diagnosed with cancer and treatment starts, the whole family is affected [4, 6]. Parents describe their lived experience of going through the child’s cancer treatment as a daily struggle in which the family’s normal daily life is disrupted and they have to focus only on the ill child [8]. It is a taxing period, and the entire family is in need of support to ease their burdens and get through the crisis [8, 9].

Though radiotherapy is a noninvasive treatment per se, it can be both stressful and challenging for children. Children are exposed to a new, unknown and highly technological environment with large radiation equipment. Additionally, the child can feel threatened by the requirement of remaining alone in the treatment room during treatment. Both of these factors can cause the child stress and anxiety [10, 11]. For both parents and children, the difficulty of understanding how radiation works, as well as its expected effects and side-effects, can provoke anxiety. Both children and parents have a need for preparation and information before radiation treatment starts [5, 12]. Previous work by Jackson et al [5] identified parents’ need for information, noting that parents were often too stressed out to absorb this information early on in the process.

The importance of parental involvement before, during and after radiotherapy must not be underestimated. Parents can re-explain information, encourage and support their children as they go through their daily treatment with radiotherapy [13]. The parents’ own emotional distress can be relieved by being involved in providing this information to the child and by seeing their child relaxed and calm rather than scared and refusing radiotherapy procedures [12, 14].

Anxiety can make it difficult or impossible for children to be left alone during treatment and, as a result, sedation and anesthesia are sometimes employed for the procedure. However, there are several advantages to radiotherapy treatment without sedation or anesthesia [11, 15]. The unsedated child experiences decreased side-effects and fewer disturbances in daily life, especially as regards sleep and nutrition. Furthermore, radiotherapy without anesthesia is less resource-intensive and expensive for the clinic [11]. Psycho-educational programs [16], audiovisual interventions [17] and hypnosis [18] have all been shown to reduce the need for anesthesia during radiotherapy for young childhood cancer patients.

Although the radiotherapy treatment process involves interactions with the most advanced technology in pediatric oncology care, few studies have investigated the child’s and the parents’ view of this particular procedure. There are, to our knowledge, no descriptive studies of children’s and parents’ experience of going through radiotherapy. There is also a paucity of studies in which mothers’ and fathers’ experiences of being with their child in the radiotherapy department are captured separately. In this study, we focus on the parents’ experience when a child who has been diagnosed with cancer undergoes radiotherapy treatment. The children’s experience of undergoing radiotherapy will be reported elsewhere.

The aim of the study was twofold: 1) to describe parents’ experience when their child undergoes radiotherapy treatment; 2) to report parents’ suggestions for improvements during radiotherapy for their children.

## Method

### Study design

The present study is the first part of a larger multicenter study aimed to improve the preparation and care environment within pediatric radiotherapy, including three of six pediatric oncology centers in Sweden. The scope of the study is to present the current state of the radiotherapy process. All curative cancer treatment in Sweden, including radiotherapy for children, is performed in accordance with national guidelines and international protocols. Each childhood case scheduled for radiotherapy is discussed with dose plans at online national rounds [19]. Accordingly, the differences in medical treatments between centers are minimized.

At each center, brief general information about radiotherapy and timing is given by physicians or nurses at the department of pediatric oncology. Parents and child are also given a small booklet containing general information about cancer therapy. At all centers, parents and child have the opportunity to visit the radiotherapy unit and meet the staff, as well as take a look at the machines and treatment room some weeks before the start of radiotherapy. One center also has a “Children’s Web” on which they can log in and have information about what happens in the radiotherapy unit and look at pictures. Detailed information about the child’s individual radiotherapy is given at the first meeting with the radio-oncologist. This meeting is usually at the radiotherapy department the day before or the same day as other preparations are made, such as skin marks, immobilization and CT for dose planning. The information is usually given to the parents and child together. Staff with long experience of meeting children and family takes care of practical information and shows the technical facilities in the treatment room, as well as the play area of the waiting room. Before the fixation, small children are given the opportunity to play with a doll and a mask on the treatment bed in the combined fixation/CT room. During preparations and treatments, the child is allowed to take a favorite doll or soft animal, as well as a CD player with music or storytelling, into the treatment room. One center also offered the opportunity to watch DVDs. Parents could be with the child during the CT assessment with the use of a lead shield. Parents always stay outside the treatment room during irradiation, but with the option of observing their child on a screen and talking with the child. At two centers, the child can have contact with parents via a thin rope. General anesthesia or sedation is given, if needed, to younger children. During the treatment period, parents and child meet the radio-oncologist and pediatric oncologist once a week to discuss physical problems and other questions about treatment or the child’s disease.

### Participants and setting

For the present interview study, a stratified sample of families was used. Eighteen families with a child from 2 to 18 years old, diagnosed with cancer and admitted to radiotherapy at one of the three pediatric oncology centers, were asked to participate in one interview each. The sample was stratified in order to include both boys and girls and to represent the three pediatric oncology centers.

Nine girls, median age of 10 (minimum 5, maximum 15), and nine boys, median age of 9 (minimum 3, maximum 15), and their parents were included in the interview study.

The children were diagnosed with acute lymphatic leukemia (n = 1), brain tumor (9), sarcoma (n = 4), neuroblastoma (n = 3), and Wilms’ tumor (n = 1). The children were receiving active treatment and they all underwent radiotherapy. No children receiving palliative care were asked to participate.

Before radiotherapy, the children had undergone chemotherapy treatment (n = 10) and tumor surgery (n = 12). Eight of the children had had both chemotherapy and tumor surgery before radiotherapy. During the radiotherapy treatment, nine of the children were on chemotherapy. Additionally, three children received anesthesia and two children received sedatives. Eleven children received radiotherapy without sedation or anesthesia.

Three parents declined participation and one parent was deceased. Interviews were performed with sixteen mothers (with a median age of 41, minimum 26, maximum 50) and 16 fathers (with a median age of 43 years, minimum 32, maximum 57). Most of the participants indicated that they worked fulltime (13 men and 8 women) and the other worked part-time and/or were on sick leave to take care of their child at the time of their inclusion in the study (n = 30, 2 missing data). Two of the participants indicated that their highest level of education was elementary school, 16 that it was high school and 11 that it was university level (n = 29, 3 missing data).

### Ethical considerations

The parents were given written and oral information about the study by a nurse at the ward when their child was admitted to the hospital for treatment. When parents agreed to participate, their names were forwarded to the first authors (GE and CÅB), who were able to provide further information about the study. The Regional Ethical Review Board in Umeå, Sweden approved the study (Ref. No.: 2012-113-31M).

### Data collection

The interviews were conducted by the first authors, GE and CÅB, both experienced pediatric nurses, from September 2012 to April 2014. The interviews were conducted at the end, or shortly after the end (within two months), of the child’s radiotherapy session. The parents decided the time and place. The interviews took place at hospitals (n = 20) or were conducted by telephone (n = 12). All interviews were audiotaped. The most common way of conducting interviews is face-to-face. Due to parents’ limited time and long geographic distances, telephone interviews were time-efficient and yielded rich data [20]. The authors were able to establish a rapport with the interviewees through initial contact to further explain the study, and the interviews were scheduled at a time and place that were convenient for them. Initially, there was some small talk about everyday things to allow parents and the interviewer to get acquainted before the interviews took place [20]. The parents were invited to speak about their experiences when their child had undergone radiotherapy. The interviews were semi-structured and started with “Can you please tell me how things are right now?” After that the parents were asked to describe their experiences of their child’s radiotherapy. Follow-up questions were asked, for example “Can you please tell me more?” and “What did you feel?” The interviews lasted 13 to 45 minutes, and they were taped and transcribed verbatim.

### Analysis

A qualitative content analysis as described by Graneheim and Lundman [21] was performed on the interview data. First, all transcribed interviews were listened to and read by the first authors, GE and CÅB, to capture the meaning and get a sense of the material as a whole. The interview text was then divided into meaning units, each comprising sentences or phrases related to the aim of the study. The meaning units were subsequently condensed, coded, compared and abstracted. Preliminary subcategories and categories were formulated. The mothers’ and fathers’ interviews were first analyzed separately in order to find out whether there were any subcategories only for either mothers or fathers; however, in each subcategory, statements from both parents emerged. Statements are presented from both mothers and fathers in the findings. The authors discussed and reflected on subcategories and categories, also together with the research group, and finally, the findings were formulated in terms of subcategories and six categories. During the analysis, an overarching theme emerged. Quotations from the transcribed text are shown in the findings [21].

## Findings

The overarching theme, that of despair turning gradually into a sense of security, with a sustained focus on and close interaction with the child, emerged. The categories and subcategories describe parents’ experience when their child undergoes radiotherapy treatment and present their suggestions for improving radiotherapy for their children (see Table 1). The categories and subcategories are presented below, with quotations from the interviews with both mothers (M) and fathers (F).

**Table 1:** Theme, category, and subcategory.

**Theme**	
Despair turns gradually into a sense of security, with a sustained focus on and a close interaction with the child	
**Category**	**Subcategory**
Cancer and cancer treatment turn life upside down and affect the entire family	Experiencing shock and overwhelming feelings
	Being anxious about the child’s health and survival
	Dealing with practical issues and sharing responsibility
	Receiving emotional and practical support from family, relatives, friends, coworkers and staff
Radiotherapy includes experiencing the child’s suffering	Experiencing the child’s physical symptoms
	Experiencing the child’s emotional distress and endurance
Radiotherapy includes experiencing intense feelings	Being close to the child and sharing similar feelings
	Feeling they are abandoning the child during the procedure
Radiotherapy includes preparation by skilled and empathetic staff	Experiencing staff having time and taking time to provide individualized information and guidance
	Experiencing having confidence in the staff
	Experiencing staff showing various degrees of empathy and consideration, and taking good care
Radiotherapy includes the experience of gradually learning to cope with the process	Experiencing getting used to the process over time
	Finding facilitating routines and coping strategies
Parents’ suggestions for improvements during radiotherapy	Requesting concrete and repeated information
	Needing individualized distractions and well thought-out procedures
	Needing routines and everyday life
	Requesting child-friendly and family-friendly staff in a child-friendly environment

### Cancer and cancer treatment turn life upside down and affect the entire family

#### Experiencing shock and overwhelming feelings

When a child has been diagnosed with cancer and has to undergo radiotherapy, parents experience shock and chaos and have to come to terms with the fact of a life-threatening illness. The feeling can be described like this: “Everything has been difficult, and the most difficult part was to digest everything—that your child is seriously ill” (0310M). Parents can experience overwhelming feelings like feeling sick and mentally exhausted, as well as uncertainty, distress and fear of the illness and the radiotherapy treatment: “Oh, it’s so terribly difficult, you’re so sad and so extremely worried and afraid” (0103M). They feel that everything concerning the disease and treatment is happening so fast that they cannot absorb the information or understand what is happening.

#### Being anxious about the child’s health and survival

Parents state that it is difficult to watch how sick the child becomes due to surgery and/or chemotherapy, and radiotherapy. Some parents report that they were prepared for their child’s radiotherapy, while others state that they are not, and that they thought the treatment would be completed with surgery and/or chemotherapy: “It was really hard, because I thought everything was done since the tumor had been removed in the operation” (0303M). All of the parents also express their fear of late effects from the radiotherapy, for example, impaired memory, damage to parts of the brain and/or the body, and mental changes. Many of them describe how they try to be positive in front of their children, but deep inside they have questions about the child’s survival and say that they are willing to let the child undergo any treatment as long as it will result in a cure. Some of them describe worries about the future: “…a little worried that it could affect the brain…my child’s future. Obviously you worry when there’s radiation involved [pauses]…that it could affect the brain, the memory, and things like that” (0205F).

#### Dealing with practical issues and sharing responsibility

A child’s illness affects the entire family. Parents must deal with many different practical issues regarding staying at the hospital when their child has treatment. They miss their home, their family and friends. They have to make arrangements for themselves and their family. They are trying to manage everyday life for the entire family. “Nowadays we don’t live together, but we’ve been together as much as possible at any rate, but not the entire time…we have two other children, so…we’ve made sure they can be wherever suits them best” (0310F). Parents often share responsibilities with each other, each one staying for a few days or a week at the hospital with the child and the next week staying at home taking care of their other children and work at home. Parents state that their ill child comes first, and they gradually learn how to be a parent of a sick child.

#### Receiving emotional and practical support from family, relatives, friends, coworkers and staff

A lot of the families stay at hospitals far from home. Parents emphasize that the time they can spend together at home as a family and the support they receive from each other in the family are essential. They describe the practical support they receive from grandparents looking after siblings and helping with emotional and practical support. Other sources of support mentioned are other people close to the family, such as relatives and friends. Coworkers and employers are described in positive terms, and their support is of great significance: “Oh, I have an amazing employer and coworkers. They’ve all said ‛Forget about us, drop by and have coffee with us whenever you want…you’ve got another job now” (0202M). Parents also feel supported by “the wonderful hospital staff” (0302M), social workers, teachers and parents of other children at the ward.

According to parents, their children get support from their siblings: “They [the older siblings] are pretty close–they do things together, try to cheer up and stay in close contact, they have the same interests” (0202M). Friends, schoolmates and teachers seem to play a significant role. The staff at the hospital school, as well as the play therapy unit, plays an important role in supporting children while they are sick.

### Radiotherapy includes experiencing the child’s suffering

#### Experiencing the child’s physical symptoms

Children undergoing radiotherapy treatment suffer from physical symptoms. The parents comment, for example, on nausea, diarrhea, headache, poor appetite, hair loss, fatigue and burns. They describe the physical changes they see in their children: “It’s like it’s not her body, you might say…The tiny, tiny feet just cannot support her weight” (0205M). Most parents describe various side-effects of the radiotherapy, and they also explain that the child can experience sensations: “He see lights flashing in his head when he gets radiation, and it makes a bad odor, an odor that isn’t in the room, of course; it’s his brain that brings it on” (0101F).

#### Experiencing the child’s emotional distress and endurance

Parents find the radiotherapy treatment difficult, depending on their child’s reactions. Young children are generally anesthetized or sedated, while older ones are conscious. For some of them, the radiotherapy session goes well: they return to consciousness and it is done. For others, however, it can be very difficult. Although they are sedated, they may be very frightened and anxious: “Oh, he was afraid someone would put a needle into him, or that it would hurt. Even if we explained to him that …it won’t hurt” (0309M).

To expose one’s child to radiotherapy each day is described as “torture” (0101M). “He doesn’t like the radiation…not fun…he gets strapped into place, you know…he thinks being strapped down is so uncomfortable…the radiation is so incredibly awful that he can’t listen to the music after because he makes him remember it” (0101F). Parents find it very hard to stand beside and observe the child’s reactions: “[whispers] Yeah, the hardest thing [sobbing while speaking]…It’s seeing that she can’t stand it. That’s the worst thing…It’s mainly the mask–it makes her panic…” (0201M). Many parents comment on difficulties with the mask: “She gets caged inside a mask that is then strapped to a table. She can’t move at all. She’s had such terrifying visions, like that she was going to fall off the table, and her head would stay on the table but her neck would be broken” (0106M). Other things that affect the experience of the radiotherapy session is that making the adjustments takes time and entails discomfort: “Being stuck there for about an hour, fighting with the mask, trying to get it right, she finds it’s so awful. And it’s stuck onto her, you know. That was probably the hardest part” (0201F). Parents of smaller children who not are able to express themselves in words observe their children and try to understand the child’s feelings: “Sometimes I ask myself what she can be thinking? What is she feeling? She is so young…and she’s gone through so much” (0310M).

### Radiotherapy includes experiencing intense feelings

#### Being close to the child and sharing similar feelings

Parents describe how they have to put everything else in life aside during treatment in order to be close to and concentrate on their child and treatment. “It’s extremely surreal—you become very close to each other when you have to stop and put everything else in your life aside” (0103M). The child’s mood and condition influence the parents’ condition. If the child’s reactions to the hospital stay and treatment are positive, parents feel relief: “When I felt my child was relaxed and feeling good after a few times, I too was able to relax a bit…I felt a little relieved too” (0310M). Parents’ and children’s feelings are intermeshed: “How hard it is for her is reflected in how smoothly things go for us too” (0102F). When the child is afraid, sad and experiencing difficult symptoms, parents describe feeling down.

#### Feeling they are abandoning the child during the procedure

Parents describe that the child must lie still and be left alone in the treatment room during radiotherapy. While the child, if a younger child, is sedated or asleep, parents’ still feel it is difficult to leave the child: “The anesthetic is working fine, but it’s hard to leave one’s child alone behind the steel doors…it feels horrible to leave one’s little three-year-old there” (0303M). The parents describe having many feelings during the procedure, such as worry, anxiety, distress and being in a vulnerable situation where they have to trust others: “…there is always worry. There is a risk with anesthesia, so it’s not a good feeling…to leave the room and see her unconscious” (0310F).

### Radiotherapy includes preparation by skilled and empathetic staff

#### Experiencing staff having time and taking time to provide individualized information and guidance

Parents report that the staff at the radiotherapy unit invited them and their children for a visit, at which they received information, asked questions, were told about what was going to happen checked out the treatment room and the equipment, and met the staff. Some children have to wear a mask during radiotherapy, and for some children, make construction and trying on the mask is a stressful, unpleasant experience: “First a visit prior…you meet the people and, and the material [the mask], they let her try it on… it gets pretty hot on the face so she was allowed to take it home and practice a bit, or play around with it to become used to it. So they were really great. Then we made a mask for her favorite stuffed animal, too” (0315F). Parents report that they have recurring questions about radiotherapy–for example, concerns about side-effects, nausea, fatigue, and late effects. Parents find it easy to ask questions and communicate with staff, and they often have the opportunity to do so while the child is getting treatment. However, some parents find the information inadequate. They ask for more information, find that what it provided is incomplete, and they want someone they can turn to when questions arise. Parents mention that their children also have questions concerning radiotherapy: “I want to mention that, and he [the child] has wondered about it—what kind of radiation is it? Radiation is dangerous: you can get cancer from radiation. Why do you not get cancer from this radiation?” (0103M).

#### Experiencing having confidence in the staff

Almost all parents report that they and their children have confidence in the staff: “I think that has worked really well and I think he has felt safe and secure–he hasn’t been particularly anxious at all” (0103F). According to parents, seeing the same staff each time can build confidence. Parents report that the staff know and prepare the kind of preparation each child prefers. Staff and the children and their parents get to know each other, and the staff adjusts details during the radiotherapy session in accordance with the child’s and parents’ wishes: “They took note of certain things. Because she didn’t want to have this kind of paper [on the hospital bed], she had to have a sheet…Then she wants it to be silent when she puts the mask on–they shouldn’t be talking” (0201M). Almost all parents mention the staff’s responsiveness to the child and their interest in getting to know the child.

#### Experiencing staff showing various degrees of empathy, and consideration and taking good care

Parents describe that they are satisfied with the care of their child and themselves. The staff is described as empathetic, thoughtful and respectful, and they take good care of their child. “When they get him in the waiting room, you get a feeling that he is the only patient they have all day…If staff can get all their patients to feel like the only one and the most important person throughout the day, there’s such a strong focus on him, it’s a really, really great feeling” (0101M). The staff also shows interest in the children and tries to see each child as an individual.

The staff’s thoughtfulness and efforts vis-à-vis the children are seen in, the details of their interactions with the children: This is described by the parents: “They asked if there was anything special he would like. They had marbles, and he decided he wanted to start collecting them, so now he gets a marble every time he has radiation” (0203M). Some parents describe that their children have a favorite member of the staff. They can give five extra minutes time to talk to, care for and become personally involved–for example, offering to bring a CD from home. Parents also describe that the staff try to do their very best for the child: “Then there are some staff who are a cut above, I guess I could say. They’re all good, but some are fantastic. Some who just go that extra mile, tuck him in a little more nicely, place the teddy bear right in beside him, give him a hug, so you feel they give that little bit extra” (0302M). Many parents describe being well taken care of and that they appreciate when staff can be in command, take over and make suggestions for how to solve problems that arise and thereby relieve parents when they do not have the strength.

### Radiotherapy includes the experiences of gradually learning to cope with the process

#### Experiencing getting used to the process over time

Parents say they get used to radiation therapy over time and can more easily cope with the situation. They adjust to the procedure, know what will happen, feel safer and understand how their child reacts: “[She]’s gotten used to the situation, but the people around her have also gotten used to it…they sort of understand the state of things” (0201F). Parents describe how some children feel better in the end, with fewer side-effects and symptoms, and some children even need less sedation because they have become accustomed to radiotherapy.

#### Finding facilitating routines and coping strategies

Planning each day and solving problems by finding routines that suit both the child and family life are essential: “A clear structure to tell me what I should do next…thinking up lots of fun tricks. Then we make sure we do them every time…the ball of yarn under the door, so we have a kind of connection, she gets to wear Mommy’s sweater, we play the right CD, have the right color on the ceiling using these bubbles…so she will feel at home” (0201F). They stay at the hospital or at home, and point out that they appreciate being able to request time for the radiation therapy. Planning activities of different kinds and entertainment after the radiotherapy helps the child get through treatment. Some parents describe that they set goals or plan activities to look forward to, such as a trip, go to a movie or other activities.

Children who are awake during the radiotherapy use a variety of distraction activities during their treatment and, for example, have their stuffed toys and listen to music or a story, or watch a film. Some children use positive thinking: “He actually thought of that himself when in the sitting radiation. He often thinks about graffiti and hockey—things that interest him” (0102M). Self-talk or having a quiet time, holding a string between child and parent, and having massage before treatment are practiced. Smaller children appreciate receiving a gift after radiotherapy as a positive reward: “He knows there are 30 radiation treatments, and he always gets to choose something [as a reward], so because there are 30 students in his class, he has decided to take one item every day. This is something he looks forward to and plans what he’s going to give each person” (0101M).

All parents comment that they know radiotherapy has to be done. They describe using acceptance and emotional detachment as coping strategies: “Somehow, you just shut it out…of course, sometimes you’re thinking ‛Golly, time for the radiation–it’s for real now” (0209M). Descriptions about avoiding thinking and talking about radiotherapy occur among the children: “The child doesn’t want us to talk about the radiation…doesn’t want to hear anything about the radiation…wants to shut it out of her thoughts as much as possible” (0101F), while others seem to accept the procedure and the situation.

### Parents’ suggestions for improvements during radiotherapy

#### Requesting concrete, repeated information

Parents express their need for information from physicians and the staff at the radiotherapy unit. They want to have repeated information about their child’s treatment, as questions arise during treatment and they want to understand what is happening: “Seeing the image from the radiation–how large a wound it produces and a little about what zone is irradiated, and so on. So you can kind of know what happens. Plus, maybe get a better explanation of what the radiation entails and what it does—what happens to all the cells” (0106F) The parents think it is important that both of them receive the same information. They recommend that other parents and children visit the treatment room to try out the equipment and meet the staff before the treatment starts. Parents suggest that their children be informed by means of an age-appropriate film, by using pictures and ensuring that their children get information about all possible side-effects. Making and testing the mask is mentioned as being problematic, and product development of the mask is suggested.

#### Needing individualized distractions and a well thought-out procedure

According to parents, suitable distractions for their children during treatment include watching a video, listening to a story told by the parent using headphones, having stuffed animals with them in the treatment room, and involving and using parents in distractions: “Find out, with the help of the child, ‘What might help make this less difficult for you?’ You can get storytelling on CDs, there’s music… I believe all kids have something …that makes difficult experiences more bearable for them” (0101M). Parents want a well thought-out, individualized procedure with no waiting time that is adapted to each child’s capabilities, limitations and needs in order to facilitate the treatment of the children.

#### Needing routines and everyday life

Parents report that they can make the radiotherapy treatment easier by finding routines for themselves and their child, whether they live at home or stay at the hospital. Their usual everyday life is of great importance when life is turned upside down and they want to continue their familiar routines. Parents appreciate living at home or close to the hospital and having their family nearby: “We had the benefit of being able to borrow one of the childhood cancer foundation’s apartments. That has been incredibly significant—it’s a little like a home. We can be together…make our own meals…we try to have a kind of summer vacation ever so often” (0106M). They also need to be able to engage in appropriate activities alongside radiotherapy to sustain them so that they will be up to their everyday obligations. They appreciate being able to have radiotherapy treatment at a time they decide themselves, as this eases the making of the arrangements and the organization of life for the child and the whole family. It is of great significance that their children can continue to go to school and visit the play therapy unit.

#### Requesting child-friendly and family-friendly staff in a child-friendly environment

Parents mention the importance of the staff’s having a welcoming manner and taking good care of both parents and children. Parents require child-friendly staff who understands the importance of adjusting language and care to the child’s age and are not in a hurry. They want the staff to be aware that preparing and adjusting the child’s position can take more time than expected. When suggesting improvements, parents return to how essential it is to have confidence in the staff: “But the most important thing is to feel confidence in the people you deal with–you’d like to advise them [other parents] to basically believe what the doctors say…they’ve told you, ‘We’re telling you everything we know,’ and that lets you let go of a lot of other worry…” (0103F). Furthermore, parents suggest child-friendly waiting rooms, with furniture and environments adjusted to children of different ages, in cheerful colors. They also suggest having more toys, games and books available for their children.

Parents describe how small gifts, such as marbles pencils or a toy, suitable for both girls and boys, given after each radiotherapy session, could make treatment easier for their children and help them to look forward to the next treatment.

## Discussion

The overarching theme, which synthesizes parents’ experience when their child underwent radiotherapy, was that despair turns gradually into a sense of security, with a steady focus on and interaction with the child. Parents described how the cancer and the cancer treatment turned their lives upside down and affected the entire family. Further, for the parents, radiotherapy included receiving patient preparation by skilled, empathetic staff, experiencing their child’s suffering, experiencing intense feelings, and learning gradually to cope with the process. Finally, parents’ suggestions of possible improvements during radiotherapy were described.

The findings reveal that children’s cancer diagnosis and treatment are stressful to the entire family. This is in line with earlier publications [4–6]. Post-traumatic stress symptoms have also been reported [22, 23]. The illness is often interpreted as life-threatening, expressed in the present study by the parents’ anxiety about the child’s survival, which is supported by other authors [4, 5]. This fact could potentially affect their own stability, and they describe benefiting from the support of healthcare staff and close friends. The need for support is well-described by others [8, 9]. Practical issues need to be resolved; including transportation to the hospital or how to take care of siblings. Otherwise the burden on the parents may prove excessive as the ill child needs comfort and care. It appears that most families in the present study shared the responsibility for their ill child and for the rest of the family. In Sweden, both parents can obtain sick leave, which may facilitate their sharing of the responsibility. If the family lived far away, being able to stay in an apartment instead of at the hospital was appreciated.

It is essential that radiotherapy be preceded by preparation of the family by skilled, empathetic staff, as proposed by parents. The information provided and the start of therapy influence how well the program overall will turn out. Every child is unique and has to be treated individually, as described by parents in the present study, and a feeling of confidence arises. Unfortunately stressed-out staff may, as described by a parent, affect collaboration and the success of the treatment procedure. It can be a challenge when on a tight schedule to interact with children and parents while maintaining a respectful approach to the child. Sometimes anesthesia has to be given to preschool children in order to ensure a correct, precise position during treatment. Preschool children are also able to cooperate if they are given time and appropriate emotional preparation, and studies in radiation settings show that it is possible. Parents of children undergoing MRIs received preparation and information about the examination [24]. Age-appropriate preparation, distractions and good communication between family and staff can make it possible for the children to undergo the examination without receiving anesthesia [24].

Parents described experiencing their child’s suffering of symptoms, emotional distress and endurance. The treatment and side-effects were sometimes very distressing, according to the parents. Pöder, Ljungman and von Essen found an association between parents’ ratings of their children’s symptom burden and their own emotional distress [25]. Studies described an association between mothers’ and fathers’ ratings of their child’s symptom burden [25, 26]. They indicate that parents have similar experiences and that both mothers and fathers are close to their child. In this study, the findings reveal that parents are closely connected with their children, and that they suffer both from their own experiences and from seeing their child suffer, as has also been reported in other studies [7, 27].

Parents experience that they have to leave the child alone in the treatment room and be separated. Feelings of anxiety when abandoning a child may occur and can be understood from the perspective of the Attachment Theory by Bowlby [28]. Healthcare staff may help parents be aware of this process, and may need further training for supporting parents in these situations. If the child reacts with anxiety, it may be more difficult to leave the child alone in the treatment room. Therefore it is necessary to find ways to make younger children feel secure by using age-appropriate information and parental closeness in other ways—for example, through a string or the parent’s voice through an intercom.

Although radiotherapy is a stressful treatment, the parents described gradually becoming accustomed to the process through the support they received from staff in the treatment room, how well the child manages the treatment, and the coping strategies described by the parents. A range of strategies are described: they find an everyday routine and structure, and that helps. Finding a goal and having activities suitable for the entire family are other strategies. Healthcare staff at the oncology ward, including staff at the hospital school, and play therapy may also help families find suitable routines. Coping strategies and adjustment, described in the present study, to the cancer diagnosis and treatment are in line with other authors’ findings [29]. What exactly helped the parents in this process requires further investigation.

Parents have suggestions for possible improvements during radiotherapy. They requested repeated information, which is also supported by previous reports [5]. A concrete, individualized approach from the staff can contribute to a well-functioning process. The visit to the radiotherapy ward was essential for the parents, as was finding a form of distraction that suited the individual child. New technology could be used to enhance the distraction. The environment is a factor that needs to be enhanced to make children less frightened and more comfortable. Parents, in expressing the desire for child-friendly staff, refer to examples showing that it is already a reality, even though it sometimes fails. To reach a family-friendly approach according to pediatric family-centered care could be an option [30].

While some of the insights gained from the interviews concern the direct experience, others point more directly to areas for improvement within the current treatment experience of both parents and their children. Indeed, parents propose various ways in which the process could be improved. While the purpose of this paper has been only to report our findings from our interviews with parents within the multicenter study, these insights will be combined with this study’s additional research into children’s and families’ needs during pediatric radiotherapy and synthesized into key opportunities for innovation in the radiotherapy treatment experience, using a human-centered design approach [31]. Our next steps will be to take our insights and turn them, in collaboration with a team of designers, into actionable interventional changes in the radiotherapy treatment process.

### Methodological considerations

To ensure the credibility of the study, parents of both genders, various ages and types of experiences of childhood cancer contributed their perspectives [21]. During the analysis, the authors moved back and forth in the research process, discussed data collection, analysis, interpretation and literature, and reflected upon the findings. [21]. Interviewing was an appropriate way to gather data and gave rich descriptions of the parents’ experience during radiotherapy and suggestions for improvement [21, 32]. The dialog during the interviews was free and unstructured, and parents were encouraged to share their experience through follow-up questions. Many parents said it felt good to share their story so that they might be able to help other parents in a similar situation, and they also pointed out that they needed more time to talk about their situation. According to Trier-Bieniek [33], telephone interviews can produce honest discussions and rich data and make it easy to talk because of the anonymity. On the other hand, the lack of body language and visual cues is a disadvantage. The authors listened attentively to the participants and were prepared to interrupt the interview if there were signs of crying or upset feelings. Some parents reacted with crying and said they felt moved during the interview, but no one needed extended support after the interview. As the parents were interested in taking part in the study, we regard the data as trustworthy.

The study contributes to our understanding of parents’ experience when their child is undergoing radiotherapy treatment. Some strengths and limitations of the study should be noted. We have interviewed both mothers and fathers in the study. The joint authors (GE, CÅB) analyzed the interviews of mothers and fathers separately. Statements from both genders occurred in each subcategory. This indicates that in Sweden the experience of mothers and fathers is similar. Separating the findings would not have yielded any further information; however, any gender differences may be easier to find in quantitative studies. The interviews with parents were conducted at the end of the radiotherapy treatment, which could be seen as a limitation. On the other hand, parents had an overview of the entire process from start to finish; moreover, the interviews with parents produced valuable data that support the present design.

We have not mentioned the ages of the children in the findings, as the parents’ experience does not always depend on the child’s age; rather, it is related to the child’s illness, treatment, side-effects and suffering. The findings can be transferred to other pediatric cancer centers in Sweden. Whether they are transferable to a broader context, we will leave to the reader to determine.

### Conclusion

Parents described a heavy burden before and around the start of radiotherapy. The overarching theme, based on the findings, that despair turns gradually into a sense of security, with a steady focus on and close interaction with the child, indicates that the parents learned gradually to cope with the radiotherapy process as the child got used to treatment and accepted the process, as well as the physical and psychological suffering. Further development of age-appropriate information and distractions were requested. Possibly, a systematic strategy for psychological preparation and training among radiotherapy staff to develop a family-friendly approach according to family-centered care could be an option to further develop the care of children and their families during the radiotherapy process.
